# *Trust Your Gut*: Galvanizing Nutritional Interest in Intestinal Cholesterol Metabolism for Protection against Cardiovascular Diseases

**DOI:** 10.3390/nu5010208

**Published:** 2013-01-16

**Authors:** Casey J. Wegner, Bohkyung Kim, Jiyoung Lee

**Affiliations:** Department of Nutritional Sciences, University of Connecticut, 216 Advanced Technology Laboratory Building, 1392 Storrs Road, Storrs, CT 06269, USA; E-Mails: casey.wegner@uconn.edu (C.J.W.); bohkyung.kim@uconn.edu (B.K.)

**Keywords:** trans-intestinal cholesterol efflux (TICE), reverse cholesterol transport (RCT), intestine, liver X receptor (LXR), peroxisome proliferator-activated receptor δ (PPARδ), calorie restriction, sirtuin, fasting, NAD(+)

## Abstract

Recent studies have demonstrated that the intestine is a key target organ for overall health and longevity. Complementing these studies is the discovery of the trans-intestinal cholesterol efflux pathway and the emerging role of the intestine in reverse cholesterol transport. The surfacing dynamics of the regulation of cholesterol metabolism in the intestine provides an attractive platform for intestine-specific nutritional intervention strategies to lower blood cholesterol levels for protection against cardiovascular diseases. Notably, there is mounting evidence that stimulation of pathways associated with calorie restriction may have a large effect on the regulation of cholesterol removal by the intestine. However, intestinal energy metabolism, specifically the idiosyncrasies surrounding intestinal responses to energy deprivation, is poorly understood. The goal of this paper is to review recent insights into cholesterol regulation by the intestine and to discuss the potential for positive regulation of intestine-driven cholesterol removal through the nutritional induction of pathways associated with calorie restriction.

## 1. Introduction

Elevated low-density lipoprotein (LDL) cholesterol in circulation can lead to cholesterol deposition and crystallization within artery walls, which is recognized as the hallmark of early atherosclerotic lesions [1]. It has been estimated that one in every six deaths in the US is attributed to coronary heart disease (CHD) [2]. As CHD continues to burden developed countries, the need for more effective strategies to lower plasma cholesterol levels becomes increasingly apparent. Current hypocholesterolemic strategies largely focus on either lowering circulating LDL cholesterol or promoting reverse cholesterol transport (RCT), a process classically thought to involve the delivery of excess cholesterol from the periphery to the liver for subsequent biliary excretion. However, recent evidence demonstrates that the intestine is host to novel pathways capable of modulating whole body cholesterol metabolism. Targeting the intestine to lower plasma cholesterol concentrations is therapeutically attractive because stimulation of hepatic cholesterol secretion to bile can facilitate gallstone formation [3,4].

The role of the intestine in the regulation of whole body cholesterol status has been historically underestimated. The recent elucidation of trans-intestinal cholesterol efflux (TICE) [5,6] and intestinal contributions to high-density lipoprotein (HDL) biogenesis [7] and RCT [8,9] make the gut an excellent nutritional target for CHD prevention. However, the metabolic states and biological processes that regulate cholesterol homeostatic pathways in the intestine are poorly understood. The goals of this review are to discuss recent understandings of the role of the intestine in the removal of circulating cholesterol and to examine the interface between intestine-specific cholesterol regulation targets and cellular responses associated with calorie restriction.

## 2. Trans-Intestinal Cholesterol Efflux (TICE)

It has long been accepted that cholesterol removal from the periphery, and subsequently the body, is confined to hepatobiliary excretion via HDL-mediated RCT. However, overlooked evidence dating back as far as the early 20th century has suggested a non-biliary route of cholesterol excretion [10,11]. Recent observations have renewed focus on this possibility [12]. Using ATP-binding cassette transporter A1 (ABCA1) knockout (*Abca1*^−/−^) mice, which have nearly undetectable plasma HDL cholesterol levels, Groen *et al.* [13] showed that a lack of HDL did not affect hepatobiliary cholesterol secretion and fecal excretion of neutral and acidic sterols. Plosch *et al.* [14] and Kruit *et al.* [15] also demonstrated that increased fecal neutral sterol loss by the activation of liver X receptor (LXR) could not be solely attributed to increased hepatobiliary cholesterol excretion. More recently, liver-specific acyl CoA:cholesterol acyltransferase 2 (ACAT2) depletion in mice paradoxically resulted in increased fecal sterol loss without increases in biliary cholesterol excretion [6]. This collection of increasingly confounding evidence has led to the recent elucidation of trans-intestinal cholesterol efflux (TICE).

TICE refers to the flux of cholesterol from circulating lipoproteins through the intestine and into the lumen for subsequent excretion or reabsorption [16,17,18]. TICE has been shown to occur throughout the small intestine with the greatest percentage of TICE occurring in the proximal intestine [5]. To gain insight into the role of HDL in TICE, HDL particles with radiolabeled cholesterol were injected into mice genetically deficient of ABCA1 and scavenger receptor class B type 1 (SR-B1), which have extremely low HDL cholesterol levels and a defect in HDL clearance [19]. Both wild-type and the double knockout mice failed to take up and secrete HDL-derived cholesterol via TICE, suggesting a minimal contribution of HDL to TICE. Although the specific cholesterol donor(s) for TICE have yet to be determined, apolipoprotein B-containing lipoproteins are likely the cholesterol donors for TICE [16]. The ability to study TICE has been enabled by innovative surgical techniques (*i.e.*, intestinal perfusions [5] and biliary diversions [20]) and genetic mouse models with drastically reduced biliary cholesterol excretion such as mice with liver-specific overexpression of Niemann-Pick C1-like 1 (NPC1L1) [20,21] and multidrug resistance P-glycoprotein 2 (Mdr2) deficient mice [15]. It has been estimated that TICE accounts for ~20%–33% of basal fecal sterol losses in both humans [22,23] and mice [15,24]. Importantly, using murine models it has been shown that the non-biliary TICE pathway can be stimulated to become quantitatively more important than hepatobiliary cholesterol removal [24,25]. Activation of LXR [14,20,24] and peroxisome proliferator-activated receptor δ (PPARδ) [25], ezetimibe administration [26] and fasting [27] are known to stimulate TICE. The therapeutic implications of TICE induction were further highlighted by the finding that TICE can participate in macrophage RCT in mice [20]. Since HDL does not directly contribute to TICE in mice [19], this finding by Temel *et al.* [20] also challenges the selectivity of HDL for macrophage-derived RCT. 

Despite evidence of TICE stimulation suggested by several studies, the precise underlying mechanisms and pathways that induce TICE are largely unknown. In addition to the identification of cholesterol donors for TICE, cholesterol transporters in the apical and basolateral membrane of enterocytes, intracellular mechanisms and mediators for cholesterol trafficking and lumenal cholesterol acceptors still need to be determined to gain better insight into the overall TICE process. The use of nutritional interventions that offer well-characterized alterations in metabolism may be a logical approach to resolve these TICE unknowns. Identification of the metabolic controls and corresponding physiological events that stimulate TICE will highlight key cellular components involved in TICE and help elucidate additional therapeutic targets. Importantly, it needs to be determined if intestine-specific intervention strategies can lead to TICE induction and subsequent cholesterol removal. Intestine-specific regulation of cholesterol removal would offer nutritional targets in an organ that bypasses many of the bioavailability concerns that plague dietary nutrients and bioactive components. Preliminary evidence described above suggests that intestine-specific strategies are a viable option for cholesterol removal and this evidence will be discussed next.

## 3. Intestinal Contributions to RCT & HDL Biogenesis

At the molecular level, synthetic agonists of LXRs are arguably the most effective activators of RCT. LXRs are nuclear receptors that transcriptionally regulate lipid homeostasis and cholesterol efflux in the liver, intestine and macrophages in response to cellular sterol levels [8,28]. Activation of hepatic LXR leads to increased biliary cholesterol excretion, while also inducing hepatic *de novo* lipogenesis [9]. Therefore, cholesterol-lowering strategies have steered away from targeted stimulation of RCT via enhanced biliary cholesterol excretion considering that this approach also promotes cholesterol gallstone disease [3] and hepatic steatosis [4]. Alternatively, recent findings have shown that intestine-specific LXR activation stimulates cholesterol transport from macrophage to feces via a process called “macrophage RCT” and circumvents the side effects of hepatic LXR activation such as increased lipogenesis [8,9]. GW6340, an intestine-specific LXR agonist, stimulated macrophage RCT by ~52% in mice [9]. Lo Sasso *et al.* [8] also demonstrated that intestinal LXR activation reduced atherosclerosis development in LDL receptor knockout mice and increased RCT in mice with intestine-specific, constitutively activated LXRα. 

The intestine also plays a critical role in the biogenesis of HDL. ABCA1, a LXR target gene expressed on the basolateral membrane of enterocytes, is a ubiquitous, membrane-bound transporter that mediates cellular cholesterol and phospholipid efflux to lipid-free or lipid-poor apolipoprotein AI (apoA-I) for nascent HDL formation [29]. Murine intestinal ABCA1 is responsible for ~30% of the steady-state HDL cholesterol in plasma [7] and increased plasma HDL cholesterol via treatment with GW3965, a synthetic LXR agonist, was completely nullified in mice lacking intestinal ABCA1 [30]. Furthermore, intestine-specific constitutive activation of LXRα in mice led to increased intestinal ABCA1 expression with a concomitant increase in HDL biogenesis [8]. Cardio-protective effects from simply increasing plasma HDL cholesterol levels has proved controversial, so a premium should be placed on HDL-raising therapies that subsequently promote cholesterol excretion [31]. In this context, intestine-specific LXR activation to increase nascent HDL formation holds great promise. 

Activation of PPARδ has also been shown to stimulate RCT via an intestine-specific mechanism of action [32,33]. In general, PPARs are a subgroup of ligand-activated transcription factors that regulate a variety of genes involved in lipid and energy metabolism [34,35]. Of the several PPAR subtypes, PPARδ is the least understood, but the physiological role of PPARδ is believed to involve glucose and fatty acid metabolism [35,36]. The recent development of PPARδ agonists has led to the discovery of increased HDL cholesterol levels and RCT upon the activation of PPARδ in insulin resistant mice [37] and monkeys [38]. Moreover, increased HDL cholesterol levels were observed in healthy human subjects when a PPARδ agonist was administered [39]. Like LXR, PPARδ has been separately implicated in intestine-specific TICE stimulation [25] and RCT [32]. However, enhanced RCT from PPARδ activation has been attributed to reduced intestinal reabsorption of HDL-derived biliary cholesterol [32]. The reduction in intestinal cholesterol absorption by PPARd activation is at least partly due to decreased expression of intestinal NPC1L1 [32,33]. NPC1L1 is a cholesterol transporter that is highly expressed on the brush border of enterocytes and it is critical for intestinal cholesterol absorption [40]. Van der Veen *et al.* [33] documented that PPARδ-induced decreases in intestinal NPC1L1 expression corresponded to increased fecal neutral sterol excretion, which persisted even when increases in HDL cholesterol levels were nullified in mice lacking ABCA1. Briand *et al.* [32] showed a similar stimulation of RCT after treatment with ezetimibe, a specific NPC1L1 inhibitor. Interestingly, ezetimibe was also shown to increase TICE via a mechanism that could not be solely attributed to its inhibitory action on intestinal cholesterol absorption [26]. 

Clearly, the intestine has emerged as a dynamic organ with tremendous therapeutic potential for lowering cholesterol. However, the cellular factors that promote atheroprotection via TICE and/or intestine-driven RCT remain elusive. As discussed above, pharmacological activation of both LXR and PPARδ has been reported to independently stimulate each of these intestinal pathways for cholesterol removal. Therefore, understanding metabolic controls that promote activation of these targets may help unveil the mechanisms for intestine-specific cholesterol removal and elucidate additional therapeutic targets for cholesterol reduction via the intestine. 

## 4. Energy Deprivation and Intestinal Cholesterol Regulation

In general, the connection between energy deprivation and the gut’s pivotal role in overall health and longevity was recently demonstrated in *Drosophila melanogaster* [41,42,43]. Intestine-specific overexpression of the *Drosophila* peroxisome proliferator-activated receptor g coactivator-1α (PGC-1α) homolog, a key regulator of energy metabolism induced by energy deprivation, delayed age-related deterioration of intestinal homeostasis and extended life span of the organism. Other studies in *C. elegans* [44] and mice [45] also support the notion that enhanced intestinal function/homeostasis increases lifespan. 

The metabolic dichotomy between fed and fasted states in the intestine may offer a useful tool to study the metabolic pathways related to TICE and intestinal contributions to whole body cholesterol homeostasis. In particular, the metabolic responses to energy deprivation are an evolutionary conserved set of highly regulated biological events that promote the mobilization of stored energy and enhance oxidative metabolism [46,47,48]. Studies on energy deprivation responses at an organismal level are often carried out using caloric restriction (CR), which is defined as a 20%–40% caloric reduction *versus ad libitum* feeding [49,50]. CR has been shown to extend lifespan and protect against age-related diseases in a number of organisms, including mammals [50,51,52]. Notably, CR has been shown to mitigate the risk for atherosclerosis development in humans [53]. The hallmark signatures of CR in mammals reflect the reciprocal activation of AMP-activated protein kinase (AMPK) and NAD^+^-dependent sirtuins (SIRTs) [48,54,55,56,57]. AMPK responds to increased cellular AMP/ATP ratios by increasing cellular NAD^+^ [56]. Interestingly, AMPK activation has been shown to mediate the CR-like effects of PPARd agonists [36]. It is well understood that cells have evolved to use NAD^+^ as a nutrient sensor and determinant of cellular metabolism and survival [58,59]. NAD^+^ levels have been shown to rise in numerous tissues in response to fasting, CR and exercise, while high-fat diets reduce the NAD^+^ levels in mice [60]. 

Several metabolic responses associated with energy deprivation/CR are mediated by NAD^+^-dependent mammalian orthologs of lower eukaryotic Sir2: SIRTs [61]. The seven mammalian SIRTs, *i.e.*, SIRT1-7, are an unusual and conserved class of deacetylases and mono-ADP-ribosyltransferases that use NAD^+^ as a co-substrate to regulate metabolic processes, including lipid metabolism [47,50]. SIRTs can regulate cellular activities by deacetylating a wide array of histone and non-histone proteins in response to cellular energy deficits [60]. SIRT1, the most studied mammalian SIRT, has been shown to regulate a number of transcription factors and proteins involved in cholesterol and lipid metabolism, including PGC-1α [48,62], PPARα and γ [47,63,64], farnesoid X receptor (FXR) [65], sterol regulatory element-binding proteins (SREBPs) [66,67], cholesterol 7α-hydroxylase (CYP7A1) [68] and LXR [28]. Significant decreases in plasma total and LDL cholesterol concentrations were observed in transgenic mice overexpressing SIRT1 [69], while SIRT1 knockout (*Sirt1*^−/−^) mice showed blunted HDL cholesterol increases after an 8-day LXR agonist (T0901317) administration [28]. However, contributions of SIRT1 to intestine-specific regulation of cholesterol metabolism remain to be determined.

### 4.1. Prolonged Fasting Stimulates TICE

A range of evidence exists that suggests many of the cellular processes associated with energy deprivation and CR may influence intestine-specific regulation of cholesterol removal. Sokolovic *et al.* [27] demonstrated increases in intestinal cholesterol levels and TICE during prolonged fasting (48 h) in mice. Similarly, increased output of biliary bile salts, phospholipids and cholesterol were also observed during fasting. As corresponding increases in fecal neutral sterols were not observed during fasting in this study, the physiological significance of increased TICE during energy deprivation in regulating whole body cholesterol homeostasis is not clear. Nonetheless, the findings highlight that energy status in the intestine is an important factor to modulate intestinal cholesterol flux. Elevated TICE during fasting may be partly explained by increases in biliary outputs of bile salts and phospholipids as their presence in the intestinal lumen has been shown to stimulate TICE by functioning as cholesterol acceptors [5]. Alternatively, Danielsen *et al.* [70] provided evidence for the role of enterocytes in fasting-induced TICE by demonstrating that apoA-I is secreted apically from enterocytes into the lumen of porcine small intestines during fasting. The study also showed bile-dependent deposition of apically secreted, lipid-free/poor apoA-I in the brush border, and microvillar apoA-I was shown to be associated with cholesterol. The authors conjecture that this deposited apoA-I is well suited to participate in TICE [70]. ApoA-I acts as a cholesterol acceptor by interacting with ABCA1 [71,72,73]. Therefore, for the microvillar apoA-I to be a functional cholesterol acceptor in the lumen of the intestine, an unknown transporter on the apical membrane of enterocytes is likely necessary to interact with apoA-I. Furthermore, the capacity of apoA-I to draw cholesterol from enterocytes to the intestinal lumen may not be quantitatively significant. Thus, the contributions of microvillar apoA-I to TICE as a cholesterol acceptor need further investigation. 

While this review discusses evidence suggesting that processes associated with energy deprivation can positively regulate intestine-specific cholesterol removal, it should be noted that increases in TICE were also observed in mice fed Western-type and high fat diets [74]. The reasons for this ambivalent regulation of TICE by energy deprivation as well as Western-type diets containing high fats and cholesterol remain to be determined. In general, TICE stimulation resulting from diets rich in cholesterol and lipids is likely related to the body’s effort to remove excess cholesterol for maintaining cholesterol homeostasis. In contrast, given that adipose tissue contains 15%–20% of the body’s cholesterol in normal individuals and more than 50% in obese subjects [75], increased cholesterol mobilization as a consequence of triglyceride hydrolysis in adipose tissue may contribute to the increased intestinal cholesterol uptake and subsequent TICE during fasting. This ambivalent behavior of TICE could be problematic for nutritional interventions, yet pursuing intervention strategies that are already known to positively influence cholesterol profiles and protect against CVD (*i.e.*, inducing energy deprivation pathways) may be a logical first-step approach.

### 4.2. PPARδ Agonists and CR

PPARδ agonists induce metabolic changes consistent with CR and prolonged exercise [35]. Following short-term exposure to a PPARδ agonist, a decrease in cellular energy status and subsequent AMPK activation were observed in human myotubes [36]. Interestingly, PPARδ is highly expressed in the intestine and PPARδ mRNA levels have been shown to increase in fasted mice [76]. Furthermore, activation of PPARδ has been shown to induce TICE without influencing biliary cholesterol excretion [25]. PPARδ agonists have been shown to promote RCT and reduce atherogenic inflammation [25,32,77,78]. Thus, it appears that PPARδ may play an athero-protective role at least in part by enhancing TICE and RCT in the intestine via the modulation of metabolic pathways similarly to CR. However, intestine-specific induction of PPARδ in relation to energy deprivation has yet to be assessed. 

### 4.3. Positive Regulation of LXR by SIRT1

Studies have shown that intestine-specific LXR activation stimulates RCT/TICE [8,14,20,24]. LXR activation is generally associated with the fed state, but recent studies have paradoxically demonstrated positive regulation of LXR by SIRT1. Li *et al.* [28] showed that LXR acetylation occurs at a single conserved lysine residue (K432 in LXRα and K433 in LXRβ) and loss of SIRT1 *in vivo* leads to decreased expression of LXR target genes. In this study, *Sirt1*^−/−^ mice had decreased plasma HDL cholesterol levels and peritoneal macrophages and hepatocytes from *Sirt1*^−/−^ mice showed a reduction in apoA-I mediated cholesterol efflux [79,80]. A recent study by Defour *et al.* [81] also demonstrated that SIRT1 deacetylates LXRα and LXRβ in human skeletal muscle. It is tempting to speculate that the surge in intestinal cholesterol levels during fasting [27] offers a scenario where active LXR, and subsequent cholesterol efflux, could be positively regulated by SIRT1 during cellular energy deficits. Regulation of LXR by SIRT1 in the intestine has yet to be addressed.

## 5. Nutritional Induction of Energy Deprivation Pathways

The intestine has emerged as an organ capable of stimulating RCT and coordinating the uptake, trafficking and efflux of circulating cholesterol, *i.e.*, TICE. The evidence discussed above suggests that metabolic pathways associated with CR/energy deprivation can similarly promote TICE and/or intestine-specific RCT stimulation. However, unlike other tissues that make significant contributions to lipid metabolism, the cellular influences of CR/energy deprivation on the intestine are poorly understood. Recent studies using transcriptomic profiling suggested that the small intestine exhibits a biphasic metabolic response to fasting that differs from the well-characterized and progressive adaptive response to fasting by the liver [82,83]. This makes sense considering the unique energetic preference for glutamine by the intestine [84]. Therefore, the influence of CR on intestine-specific cholesterol removal pathways needs to be assessed. 

CR-mimetics and NAD^+^ precursors are an attractive means to study metabolic pathways that are triggered by energy deprivation in the gut. For instance, NAD^+^ precursors such as nicotinamide mononucleotide (NMN) and nicotinamide riboside (NR) hold great nutritional promise for lowering plasma cholesterol by increasing NAD^+^ and subsequently activating pathways associated with CR/energy deprivation via SIRT1 [85,86]. Dietary supplementation of NR, a compound found naturally in milk, was shown to prevent diet-induced obesity and significantly reduce plasma total cholesterol levels in C57BL/6J mice fed a high fat diet [85]. Similarly, daily *i.p*. injections of NMN (500 mg/kg) for 11 days ameliorated high fat diet-induced hypercholesterolemia in age-induced Type 2 diabetic mice [86]. Due to these findings, speculations have begun to circulate regarding the unclear mechanisms behind niacin’s potent hypolipidemic effects. Niacin, in the form of nicotinic acid, is another NAD^+^ precursor and potent serum cholesterol-lowering agent that exhibits cardioprotective effects [87]. The original observation concerning the cholesterol-lowering effect of nicotinic acid by Altschul *et al.* [88] noted that similar cholesterol-lowering effects were not observed with nicotinamide treatments. Interestingly, nicotinamide, a product of SIRT deacetylation reactions, is a known feedback inhibitor of SIRT activity [89]. Overall, NAD^+^ precursors such as NR and NMN are promising candidates for cholesterol-lowering strategies that do not produce flushing, a severe side effect of nicotinic acid [85]. Yet, the effect of NR and NMN on intestine-specific cholesterol regulatory pathways and corresponding protection against CVD has yet to be assessed. 

The red wine polyphenol and CR mimetic, resveratrol has proved to be a small-molecule activator of AMPK and SIRT1 *in vitro* and *in vivo* [90,91]. A number of recent studies have truly begun to unravel the mechanistic influences of resveratrol [92,93]. Human trials have demonstrated mixed results concerning the effect of resveratrol on various metabolic and lipid parameters [94,95]. However, the specific role of resveratrol in the regulation of intestine-specific lipid/cholesterol metabolism and fecal sterol excretion remains to be determined. Additionally, a number of other dietary polyphenols have been suggested to regulate SIRT1 and the pathways associated with CR [96]. Future research into intestinal SIRT1 modulation by polyphenols could help elucidate the mechanism underlying hypocholesterolemic mechanisms of action for many of these highly consumed bioactives. 

## 6. Conclusions

In support of the overall health promoting prominence of the intestine, recent findings have unveiled the critical roles of the intestine in the regulation of whole body cholesterol homeostasis particularly by enhancing TICE and RCT. For the field of nutrition, the discovery of novel cholesterol regulatory pathways in the intestine undoubtedly offers an unexplored platform for intestine-specific intervention strategies to lower blood cholesterol levels for protection against CVD. 
